# Edge computing based real-time *Nephrops* (*Nephrops norvegicus*) catch estimation in demersal trawls using object detection models

**DOI:** 10.1038/s41598-024-60255-8

**Published:** 2024-04-25

**Authors:** Ercan Avsar, Jordan P. Feekings, Ludvig Ahm Krag

**Affiliations:** Section for Fisheries Technology, Institute of Aquatic Resources, Technical University of Denmark, Hirtshals, Denmark

**Keywords:** Computer science, Marine biology

## Abstract

In demersal trawl fisheries, the unavailability of the catch information until the end of the catching process is a drawback, leading to seabed impacts, bycatches and reducing the economic performance of the fisheries. The emergence of in-trawl cameras to observe catches in real-time can provide such information. This data needs to be processed in real-time to determine the catch compositions and rates, eventually improving sustainability and economic performance of the fisheries. In this study, a real-time underwater video processing system counting the *Nephrops* individuals entering the trawl has been developed using object detection and tracking methods on an edge device (NVIDIA Jetson AGX Orin). Seven state-of-the-art YOLO models were tested to discover the appropriate training settings and YOLO model. To achieve real-time processing and accurate counting simultaneously, four frame skipping ideas were evaluated. It has been shown that adaptive frame skipping approach, together with YOLOv8s model, can increase the processing speed up to 97.47 FPS while achieving correct count rate and F-score of 82.57% and 0.86, respectively. In conclusion, this system can improve the sustainability of the *Nephrops* directed trawl fishery by providing catch information in real-time.

## Introduction

Demersal trawling is globally a conventional fishing method that involves towing one or more nets along the seafloor to catch demersal, or bottom-dwelling, fish and shellfish. This method of fishing is used to target a large range of species globally and in the greater North Sea species such as cod (*Gadus morhua*), haddock (*Melanogrammus aeglefinus*), plaice (*Pleuronectes platessa)*, and *Nephrops (Nephrops norvegicus)*. Demersal trawling is often considered a controversial fishing method, as it has geotechnical impacts on the seafloor that can affect the in- and epifauna present on the seabed and may result in the unintentional capture of non-target species and sizes, known as bycatch^1–3^.

Extensive efforts have been made to minimize these negative impacts, particularly to improve the selectivity of demersal trawls to reduce bycatch^4–6^ and develop gear with a reduced benthic impact^7,8^. However, such developments do not address another problem related with demersal trawling, which is having no information about the catch composition and catch rates until the end of the trawling operation. Having such information in real-time can also contribute to reducing demersal trawling’s negative impacts by ensuring that fishing quotas are fished more efficiently and better match the quota compositions available^9,10^. In other words, there is a critical lack of information as streaming of live catch data from the trawl during fishing would enable the fisher to search for more suitable grounds (e.g. avoiding grounds with high presence of juvenile individuals or little commercially sized individuals). This could eventually allow for more targeted, efficient, and sustainable fishing by reducing the bycatch and optimizing the catch rates by only fishing where it economically and ecologically makes sense to do so. This is something which can facilitate substantial improvements for fisheries targeting species like *Nephrops* which are only available to the fishery when they are out of their burrows^11^. In addition, the small mesh sizes employed in the fishery can lead to high amounts of discards^12^. Therefore, the *Nephrops*-directed fishery requires detection of *Nephrops* catch items for improving its efficiency and sustainability.

Recent studies collecting in-trawl data using underwater cameras make the visual data available onboard the vessels in near real-time^13^. This, however, brings about the necessity of processing the video in real-time to report the instantaneous catch compositions and rates in the trawl directly to the operator. Such a catch reporting system can provide a more detailed picture of what is taking place during the fishing operation. Subsequently, it can be used to make real-time decisions during trawling and eventually open a way for more sustainable and profitable demersal fisheries. State-of-the-art deep learning models developed for computer vision applications are suitable tools for automated processing of these videos. In particular, object detection methods such as You Only Look Once (YOLO)^14^ and Faster region-based convolutional neural networks (RCNN)^15^ can generate bounding boxes around the objects of interest in the video frames. The approaches based on deep learning are known to be very efficient in various marine-related applications such as recognition of fish species^16^, ecosystem monitoring using underwater videos^17^, and electronic monitoring with onboard videos^18^.

There are some studies where the catch rates are automatically determined in underwater videos. For example, Allken et al. used an object detection model based on RetinaNet to identify pelagic and mesopelagic fishes^19^. For counting the fishes, no tracking step was utilized. Instead, a linear regression model was used to estimate total catch items given the total number of fish detections in the video. In another study, an instance segmentation method, Mask R-CNN, was used to detect round fish and flat fish in addition to *Nephrops*^20^. In that study, different image augmentation methods were used to improve the detection and tracking of the fish in the videos. However, these works do not include any results regarding the processing speed of the algorithms. In the area of underwater image processing, Deep Vision, an in-trawl stereo camera system, has been utilized in some recent studies^21–23^. The major drawback of that system is that the collected data is stored in a hard drive during trawling. Hence, it does not have the feature to stream the data onboard. In addition, the studies using the data collected by Deep Vision have objectives like fish size measurement^24^, species identification^25^, and fish counting^19^ in individual frames with no concern of real-time processing. One recent example where real-time processing speed was considered is the approach by Avsar et al.^26^. The study shows the suitability of the YOLOv4 model for real-time counting of *Nephrops*. However, the results reported in that work are obtained on a high-performance computer that is not possible to access remotely during fishing. Therefore, there is a need for evaluating the feasibility of deep learning-based object detectors and edge computing hardware for real-time processing of underwater videos.

Deep learning models typically require huge amounts of computation to make predictions on the input data. Therefore, the delay observed at the output is too large to be considered as real-time processing unless the hardware is sufficiently powerful. One solution to this problem is cloud-based computing where data is transmitted from the source to a remote computer and the processing results are sent back to the source through an internet connection. However, this solution is not feasible for real-time applications as it will introduce network-related delays in the pipeline. An alternative to cloud-based approaches can be utilization of edge devices that are typically compact sized computing hardware designed to perform data processing at a point closer to the data source. Edge devices can address the related requirements simultaneously using their specialized hardware platforms that are optimized for deep learning computations while minimizing power consumption. Therefore, they are often used in deep learning applications to perform real-time data analysis and decision-making at the point of data capture, rather than sending the data to a remote server for processing^27^. This can provide several advantages, including reduced latency, improved privacy and security, and reduced bandwidth. As a result, utilization of edge devices may be critical for real-time processing of the video data onboard and eventually provide fishers with instant catch information.

The aim of this work is to investigate if the catch information can be made available to fishers in real-time using equipment that can be installed onboard fishing vessels. For this purpose, exhaustive experimentations on state-of-the-art object detection models and an edge computing device have been carried out to understand the possibility of achieving real-time speed in processing of the underwater footage collected by an in-trawl camera. In other words, performances of object detection models have been evaluated for different settings and ways to improve the overall processing speed of the video frames have been investigated. At the deployment stage in real-world applications, there may be hardware-related constraints such as buffering the inflow of frames and decrement in the processing performance of the hardware as a result of heating. Such constraints have not been included in the scope of this study which considers processing speeds of the individual frames for determining the overall performance.

In particular, *Nephrops* individuals in the videos are counted to estimate the number of *Nephrops* caught during the trawling operation. For this purpose, two of the most recent YOLO versions, YOLOv7 and YOLOv8, are used for detecting the *Nephrops* instances in the video frames. For tracking the detections in the frames, Simple Online Realtime Tracking (SORT) is used and the tracks satisfying certain conditions are considered as *Nephrops* catches. To determine the optimum experimental settings for real-time counting of *Nephrops,* the performances of numerous experimental settings are compared. For training of the object detection models, these settings include different optimizers, batch sizes, and input image dimensions. Since the processing speed is a critical evaluation criterion in this study, the effects of skipping frames in different amounts on the counting performance have been evaluated. The frame skipping operation is expected to cause degradation in the tracking performance, but it enhances the overall processing speed. To address this tradeoff, an adaptive frame skipping idea, which decides whether to skip the next frame based on the content of the frame, is proposed. Finally, the change in the frame processing speed as a result of power consumption limitation of the edge device has been observed. It is obvious that limited power consumption means slower processing of data. However, it is important to understand the rate of change in the processing speed for specific power ratings because this information is useful when the edge device is powered by an external battery. In practice, it is never possible to deliver the results in exact real-time (i.e. with no delay) because of the delays introduced at every single step of the pipeline from video acquisition to visualization of the results. Therefore, the term *real-time* used throughout this manuscript actually refers to *near real-time* and any processing speed higher than the frame rate of the video is defined as *real-time processing*. The research questions listed below are addressed in this study:

- Is it possible to achieve close to real-time processing speed of *Nephrops* catch count estimation in demersal trawl fisheries using the state-of-the-art object detectors and an edge device?
- How much does the frame skipping degrade the counting performance of *Nephrops* while improving the average processing speed?
- Is it possible to improve the frame skipping idea by modifying it to skip frames adaptively?
- What are the training and test settings for YOLO-based object detectors to obtain real-time processing with a maximized performance in counting of the *Nephrops* catches?
- How is the overall processing speed affected when the maximum allowed power consumption of the edge device is reduced?

## Materials and methods

### The dataset

The datasets used in model training and evaluation of the methodology are generated from the videos recorded on June 27, 2020, on commercial *Nephrops* grounds in Skagerrak using an underwater stereo camera system^13,20^. The YOLO models require images with bounding box annotations for training. Therefore, a dataset of 4044 images was generated from the videos recorded during the hauls. The images in the dataset were selected according to the presence of *Nephrops* or other catch items. 12.5% of these images were randomly selected as a test set and the remaining proportion was increased by 1000 additional images generated using copy-paste augmentation to form the training set^28^.

On the other hand, the ultimate performance assessment of the methodology should be performed on videos, rather than images, to evaluate overall processing speed and *Nephrops* counting accuracy. For this purpose, five videos that do not have any Nephrops objects in common with those in the training set were selected. Each video has a different duration, varying *Nephrops* ground truth counts, and different object densities, providing diverse scenarios that better represent the cases in real applications^29^. More information on the videos is available in Table 1. Since the performance of the proposed method depends on the *Nephrops* distribution throughout the videos, frame-based details (such as ratios of frames with *Nephrops* and ranges of frame numbers for each *Nephrops* presence) for the test videos are provided in the Supplementary Information file. The stereo camera was set to record videos with a resolution of 1280 × 720 pixels at 60 frames per second (FPS), and only the videos from the right camera were processed. For benchmarking purposes, both the image and video datasets are the same as those used in Avsar et al.^26^.

**Table 1:** Details of the videos.

	Duration (min:sec)	Total *Nephrops* (no.)	*Nephrops*/s	FPS
Video 1	00:55	4	0.0727	60
Video 2	01:31	6	0.0659	60
Video 3	07:30	36	0.0800	60
Video 4	08:10	40	0.0816	60
Video 5	06:29	23	0.0591	60

### Deep learning models for *Nephrops* detection

The two very recent versions of YOLO, YOLOv7 and YOLOv8, are used in this study to perform the object detection task. YOLO uses a single-stage object detection approach, hence the models of YOLO family are known to be fast and accurate. In general, YOLO architectures have the major building blocks of backbone, neck, and prediction head. YOLOv7 uses novel computational units in its backbone called extended efficient layer aggregation network (E-ELAN)^30^. E-ELAN units enable improved learning through expand, shuffle, and merge cardinality operations in their structure while keeping the original gradient transmission path. In addition, it features module re-parameterization meaning that some sets of model weights are averaged for enhancing model performance. In the prediction head of YOLOv7, an additional auxiliary head is introduced to assist in the training operation, and eventually achieve better predictions by the lead head. The technical details of the experiments and ablation studies on ultimate model structure are available in the YOLOv7 paper^30^. A tiny version of YOLOv7, YOLOv7-Tiny, was also developed to be used on edge GPU devices. Different from the YOLOv7 model, YOLOv7-Tiny uses the Rectified Linear Unit (ReLU) as the activation function and it possesses a smaller number of computational layers which yields a reduction in the number of parameters as well.

In the backbone of YOLOv8, a cross-stage partial (CSP) network that allows concatenation of the features from different hierarchical levels is used. Usage of anchor boxes with predefined aspect ratios has been a bottleneck for both speed and accuracy for YOLO models. YOLOv8 eliminates the need of the anchor boxes at the prediction phase by detecting the object center directly. As another improvement, it involves specific convolutional units called C2f modules enabling a better gradient flow during learning. The number of residual connections in the Cf2 modules, as well as the number of channels in the intermediate convolutional layers, can be adjusted by depth and width multipliers, respectively. In other words, these two hyperparameters are useful for customizing the feature extraction capability and the number of parameters of the YOLO model, which makes it possible to adjust the processing speed and detection accuracy of the model. Therefore, in the public repository of YOLOv8, five different versions have been made available; nano (n), small (s), medium (m), large (l), and extra-large (xl). YOLOv8 can be defined as an improved version of YOLOv5, however, none of these methods have an associated paper where the model details are explained^31^.

The trained models have been applied to the individual frames of the test videos sequentially to find the bounding boxes of the *Nephrops* objects. The bounding box information is input to the tracking algorithm before processing the next frame.

### Tracking and counting of the *Nephrops*

The detection of *Nephrops* individuals alone is not sufficient to be able to determine catch rates (counts) in the trawl. Therefore, these detections should be tracked throughout the video and the tracks satisfying certain conditions should be considered as catch items. It was shown earlier, that checking the tracks generated by the Simple Online and Real-time Tracking (SORT) algorithm against three different cases related to the bounding box coordinates achieves a promising counting performance^26^. Therefore, the same strategy has been followed in this study. However, for completeness, it is mentioned here as well.

**Figure 1:**
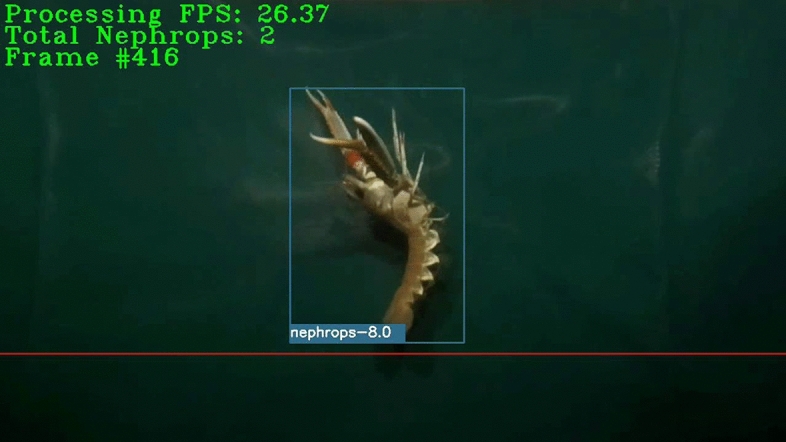
A sample processed frame showing the horizontal level (red line), bounding box and some information about the status of the processing.

The *Nephrops* tracks were generated using SORT, a computationally light algorithm for tracking objects in 2D that uses a Kalman filter to predict the states of the tracks for the next frame^32,33^. However, not all the tracks account for true *Nephrops* catches because the SORT algorithm may lose the track of the *Nephrops* in the video or *Nephrops* may swim in the opposite direction of trawling after floating for while in the field of view of the camera. To determine tracks corresponding to true *Nephrops* catches, a horizontal level is introduced at the top 4/5 of the frame height, where the position of the bounding boxes relative to the horizontal level determine whether the individual is recorded or not (Fig. 1). In particular, tracks are considered as a true *Nephrops* catch when one of the following conditions are satisfied:

i. When the bottom of the bounding box crosses the horizontal level.
ii. When the center of the bounding box crosses the horizontal level.
iii. When the height of the bounding box is greater than 2/3 of the frame height.

### Details of the training and test time settings

Efficient training of the model is essential for accurate detection of the *Nephrops* individuals. This affects the overall assessment of the system performance during testing that involves tracking and counting of the *Nephrops* in the videos. Therefore, numerous settings have been experimented with to understand how the counting performance and the processing speed changes as functions of these settings.

As mentioned earlier, the *Nephrops* detection step has been tested with YOLOv7 and YOLOv8 models separately. In addition to regular YOLOv7, its lightweight version YOLOv7-Tiny has been used for detection. As for YOLOv8, five variants with different scales and computational loads have been used. As a result, a total of seven models are involved in the detection step. It is possible to train these models using different optimizers, batch sizes, and image dimensions, each of which influences the training performance and consequently the weights of the output model. Stochastic gradient descent (SGD)^34^ and Adam^35^ are the two optimizer functions considered. Batches of 32 or 64 images were randomly generated for the model training by resizing the input images to dimensions 256, 416, or 640 pixels. These options allowed for obtaining 12 different combinations for training settings and all seven models were trained with each of these combinations, which amounts to training of 84 different models. The training operation continued for 200 epochs and the weights achieving the highest mean average precision were used during the test phase. For the remaining model parameters, default settings and values provided in the related repositories are used^31,36^.

**Figure 2:**
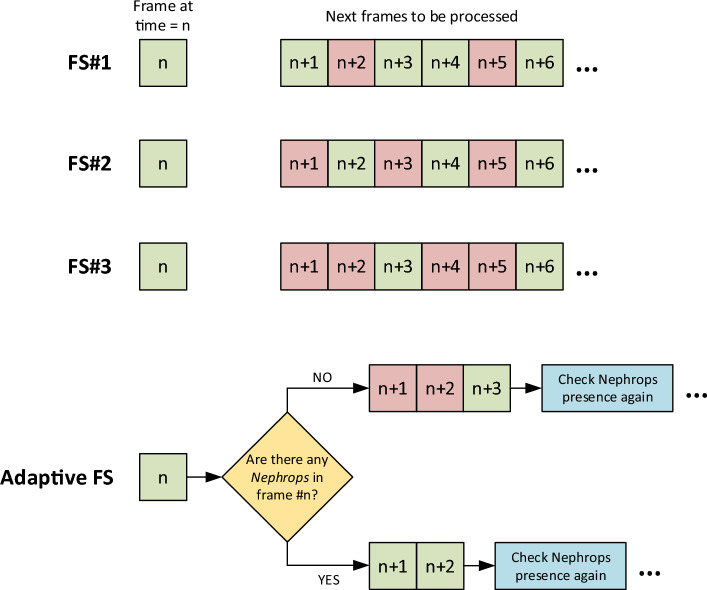
Illustration of frame skipping approaches. Red and green boxes represent skipped and processed frames, respectively.

During the testing of the models, the videos mentioned in Table 1 were processed and their counting performance assessed together with the frame processing speed. The processing of the videos was accomplished by dealing with the frames individually (i.e., one by one with a batch size of 1 frame). Prediction of the bounding boxes in the frames was done with a confidence threshold value of 0.5. Since the frame processing speed has a critical value for this study, improvements in processing speed have been prioritized at a cost of sacrificing the correct counts to some extent. For this purpose, four different frame skipping ideas have been implemented and their effects on the *Nephrops* counting performance and average processing speed evaluated. Three of the frame skipping approaches skip some intermediate frames according to predefined settings. The fourth one uses the detection output of the model to determine whether to process or skip the next frames. Therefore, the fourth approach is called adaptive frame skipping. Details of these frame skipping ideas are given below and illustrated in Fig. 2.

i. *Frame skipping #1 (FS#1)* Skip every third frame and process the others.
ii. *Frame skipping #2 (FS#2)* Skip every second frame and process the others.
iii. *Frame skipping #3 (FS#3)* Skip every second and third frames and process the others.
iv. Adaptive frame skipping: Determine whether to process or skip the next frames according to the content of the current frame. If there are no *Nephrops* in the current frame, skip the next two frames and process the third one. Else, process the next two frames. Check the presence of *Nephrops* in every processed frame and implement the same condition until the end of the video.

In particular, the first three frame skipping approaches are expected to affect the overall processing speed in a positive way while degrading the counting performance to some degree. The purpose of the adaptive frame skipping is to resolve this issue by processing the frames more often whenever a *Nephrops* is detected in the video. As a result, it is aimed to achieve higher overall FPS values and counting performance simultaneously.

In addition to the counting performance and the processing speed, it is also important to consider the power consumption of the edge device because such hardware may be required to run in remote locations with limited power resources. In the case of *Nephrops* fisheries, the next design stage may be to process the videos underwater without streaming them to an onboard station. This implies powering the edge device with an external battery that should last at least until the end of the haul (typical haul durations in the *Nephrops* fisheries range from 4 to 6 h). Therefore, optimizing the power consumption is necessary for effective utilization of the power resources and determine the battery requirements. In order to understand how the lowered power consumption of the edge device affects the frame processing speed, the same experiments have been repeated after introducing a restriction on the power consumption of the edge device. For this purpose, the edge device was first used in max power mode which may consume up to 60 W^37^. Next, an upper limit of 50 W was introduced for allowed power consumption and changes in the frame processing speed observed. Note that such a limitation does not affect the counting performance.

In summary, seven different models are included in this study, where each model has been trained with various combinations of optimizers, input image sizes, and batch sizes. During testing, four different frame skipping ideas were implemented together with the case where all frames were processed (i.e. no frame skipping). In addition, the same experiments were performed after changing the power mode of the edge device.

### Specifications of the coding environments

For training of the models, a Tesla A100 GPU with 40 GB RAM that is available at high performance computing clusters of Technical University of Denmark was used together with cudnn v8.2.0.53 and CUDA v11.3^38^. The training codes were written with Python v3.9.11 utilizing PyTorch framework v1.12.1 and torchvision v0.13.1.

All the trained model files were transferred to NVIDIA Jetson AGX Orin developer kit, the state-of-the-art edge device used for performing the experiments involved in this study. This is a single board computer optimized for deep learning applications containing 64 GB memory, 2048-core NVIDIA Ampere architecture GPU with 64 Tensor Cores operating at 1.3 GHz, and 12-core ArmCortex CPU with a maximum frequency of 2.2 GHz^39^. On the edge device, all the codes were written in Python v3.8.10 using PyTorch v1.14.0 and torchvision v0.15.0. The GPU support was accomplished through cudnn v8.6.0 and CUDA v11.4.19.

### Performance evaluation metrics

The performance metrics considered within the experiments are categorized under two groups; *counting performance* and *processing speed*. For evaluating the counting performance, each *Nephrops* track counted by the algorithm is labelled as either a true positive (TP) track or a false positive (FP) track after comparing them with the ground truth (GT) tracks. Furthermore, those *Nephrops* that are visible in the video but not counted by the algorithm are labelled as false negative (FN) tracks. The number of true positive tracks is important to assess the rate of the correctly counted *Nephrops*. Therefore, the first counting performance metric, namely the correct count rate, is defined as

$$Correct\, Count \,Rate=100\times \frac{TP}{GT}.$$

False positive and false negative tracks are two important types of counts that influence the overall performance of a method. However, these numbers are not used when calculating the correct count rate. Therefore, the F-score that considers true and false counts together is calculated as the second metric using the formula below.

$$F{\text{-}}score=\frac{TP}{TP+0.5\times \left(FP+FN\right)}.$$

As for the processing speed evaluation, the time taken to process each individual frame is recorded and its reciprocal is calculated as the processing speed in frames per second (FPS). The total processing time for an individual frame contains durations for detection and tracking together. Minimum, maximum, and mean FPS values for all the frames in a test video are reported to determine the suitability of the method for real-time applications. In case of frame skipping, the FPS value of an individual frame is multiplied by a factor equal to one more than the number of skipped frames and the result of this multiplication is recorded as the FPS value for that frame. For instance, if it takes 50ms for a frame to be processed with FS#2, the corresponding FPS value for that frame will be calculated as 40. This value is obtained by multiplying 20, the original FPS value, by 2 because only one frame is skipped before the processed frames.

Intermediate metrics, such as detection performance of the YOLO models on the test set and tracking performance of the SORT algorithm, have not been considered in this study because they are not directly reflecting counting performance. In addition, these details are already provided in an earlier study where other versions of YOLO were used^26^.

## Results

Due to the diversity in the training-time and testing-time settings, a large amount of performance metrics were produced. These results have been collected under certain topics and presented in the following subsections to enable a convenient discussion. In particular, the models generated by different training-time settings have been compared for the case when there is no frame skipping (“Overview of the models in terms of counting performance and processing speed” and “Quantitative results for selected models” section). The effects of changing the frame skipping options and the power mode of the edge device are presented separately (“Performance with frame skipping” section).

### Overview of the models in terms of counting performance and processing speed

**Figure 3:**
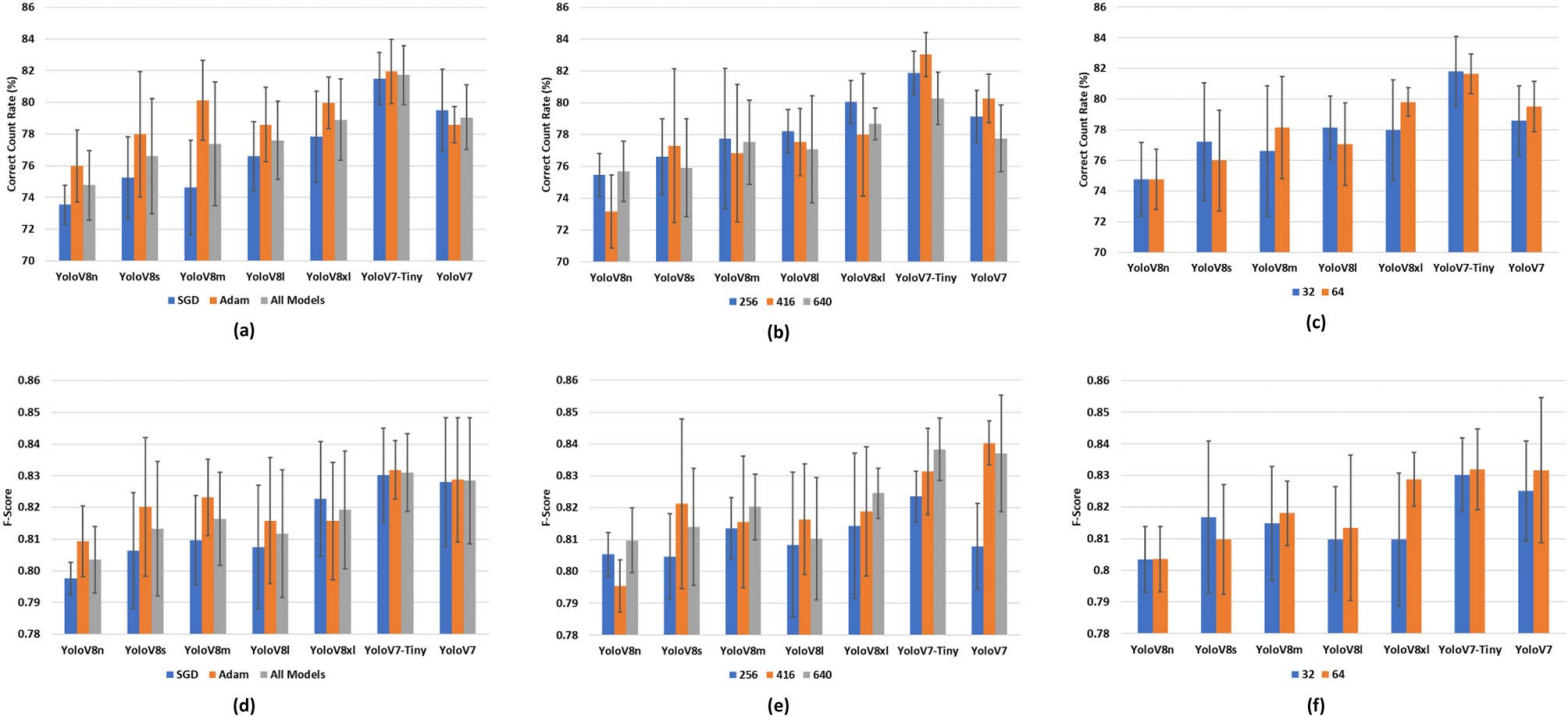
Mean and standard deviations of correct count rates (**a–c**) and F-scores (**d–f**) for different optimizers (**a,d**), input image dimensions (**b,e**), and batch sizes (**c,f**).

**Figure 4:**
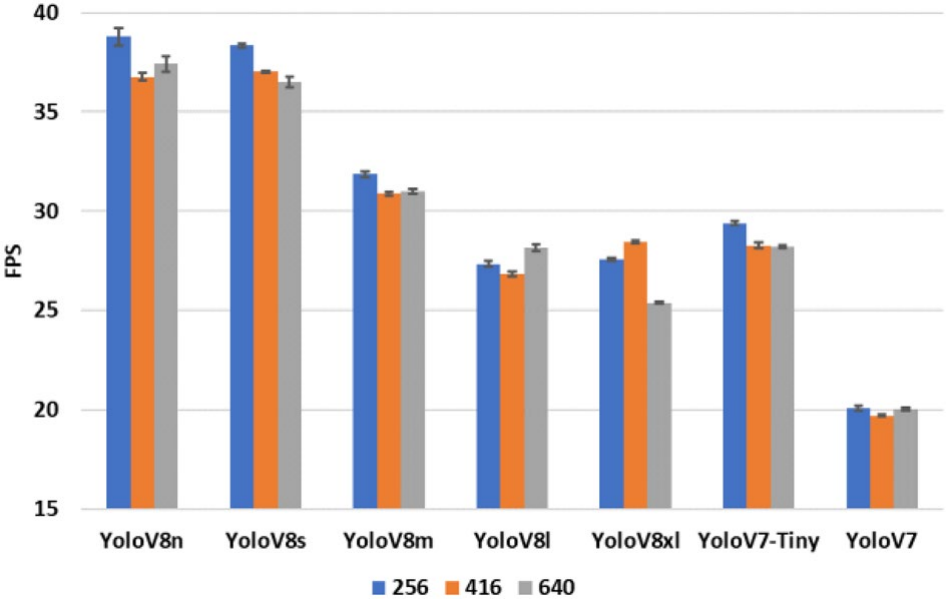
Mean and standard deviations of FPS values for different image sizes and YOLO models.

It is possible to identify the most suitable training settings by observing the performances of the trained models. For this purpose, results related with different optimizers, input image sizes, and batch sizes have been summarized by calculating their mean and standard deviations over all trained models for each of the seven YOLO models. For example, when comparing the SGD and Adam optimizers, the average correct count rates for all YOLOv8n models trained using SGD and Adam are calculated separately. This is repeated for all models, and the standard deviations are computed in the same way. In order to compare effects of the input image sizes and batch sizes on the performance, the same approach is used for different image sizes and batch sizes. This approach allows for generating a number of charts that summarize, enable visual comparison, and generate an overview of the results. For this purpose, six bar charts have been generated showing the mean and standard deviation of the correct count rates (Fig. 3a–c) and F-scores (Fig. 3d–f) for SGD and Adam optimizers, input image dimensions of 256, 416, and 640, and batch sizes of 32 and 64. Since the input image size is the only setting that affects the prediction speed of the model, FPS values for comparing the image sizes are provided separately (Fig. 4).

### Quantitative results for selected models

As a result of the experiments, an extensive number of performance measures have been generated. For simplicity and clarity, those models that perform best are investigated further. According to the results given in Fig. 3a–c, a training scheme involving Adam optimizer with an input image dimension of 416 pixels and batches of 32 images achieves high counting performance in general. As for the speed performance, YOLOv8n, YOLOv8s, YOLOv8m, and YOLOv7-Tiny are the four fastest model types, with no significant difference in counting performance than those obtained by the other three models (Figs. 3, 4). Therefore, quantitative performances of these four models trained with the aforementioned settings are provided (Table 2). In particular, the number of *Nephrops* counted by each of the models, together with the number of true positive, false positive, and false negative tracks are reported for each individual test videos (Table 2). These numbers allow for computation of counting performance metrics using the formulae given in “Performance evaluation metrics” section.

**Table 2:** Counting performances of the selected models trained with Adam optimizer, 416 pixels of input image dimension and batches of 32 images.

	Video-1	Video-2	Video-3	Video-4	Video-5	Total
Ground truth	4	6	36	40	23	109
YOLOv8n						
Output	4	4	36	22	23	89
True positives	3	4	32	19	20	78
False positives	1	0	4	3	3	11
False negatives	1	2	4	21	3	31
Correct count rate (%)	75.00	66.66	88.89	47.50	86.96	71.56
F-score	0.75	0.80	0.89	0.61	0.87	0.79
YOLOv8s						
Output	3	4	36	39	24	106
True positives	3	4	33	31	22	93
False positives	0	0	3	8	2	13
False negatives	1	2	3	9	1	16
Correct count rate (%)	75.00	66.67	91.67	77.50	95.65	85.32
F-score	0.86	0.80	0.92	0.78	0.94	0.87
YOLOv8m						
Output	3	4	36	33	22	98
True positives	3	4	32	27	21	87
False positives	0	0	4	6	1	11
False negatives	1	2	4	13	2	22
Correct count rate (%)	75.00	66.67	88.89	67.50	91.30	79.82
F-score	0.86	0.80	0.89	0.74	0.93	0.84
YOLOv7-Tiny						
Output	4	5	41	40	24	114
True positives	3	4	34	30	20	91
False positives	1	1	7	10	4	23
False negatives	1	2	2	10	3	18
Correct count rate (%)	75.00	66.67	94.44	75.00	86.96	83.49
F-score	0.75	0.73	0.88	0.75	0.85	0.82

These models are also compared in terms of their related frame processing speed, which is a critical measure for the intended real-time operation (Table 3). On average, the fastest and slowest models are YOLOv8s and YOLOv7-Tiny, respectively. The majority of (68 out of 84) the models are capable of achieving counting performance above 80%, which can be considered as providing sufficiently valuable information to the vessel during fishing. However, the average processing speed of the frames are not high enough for delivering the information in real time (Table 3). Therefore, the same experiments have been carried out by skipping frames in different amounts as explained in “Details of the training and test time settings” section.

**Table 3:** Frame processing speeds of the selected models in frames per second (mean[min–max]).

	Video-1	Video-2	Video-3	Video-4	Video-5	Average
YOLOv8n	36.49 [20–42]	36.26 [30–39]	36.94 [23–41]	36.73 [27–42]	37.08 [30–40]	36.70 [26.0–40.8]
YOLOv8s	**36.83 **[22–42]	**36.32 **[32–39]	**37.16 **[27–42]	**37.29 **[26–43]	**37.84 **[29–43]	**37.09 **[27.2–41.8]
YOLOv8m	30.33 [22–32]	30.75 [24–34]	31.19 [22–35]	31.15 [24–33]	31.15 [25–33]	30.91 [19.4–33.4]
YOLOv7-Tiny	28.12 [17–30]	28.00 [18–30]	28.15 [21–31]	28.25 [22–31]	28.24 [20–31]	28.15 [19.6–30.6]

Best values are in bold.

### Performance with frame skipping

**Figure 5:**
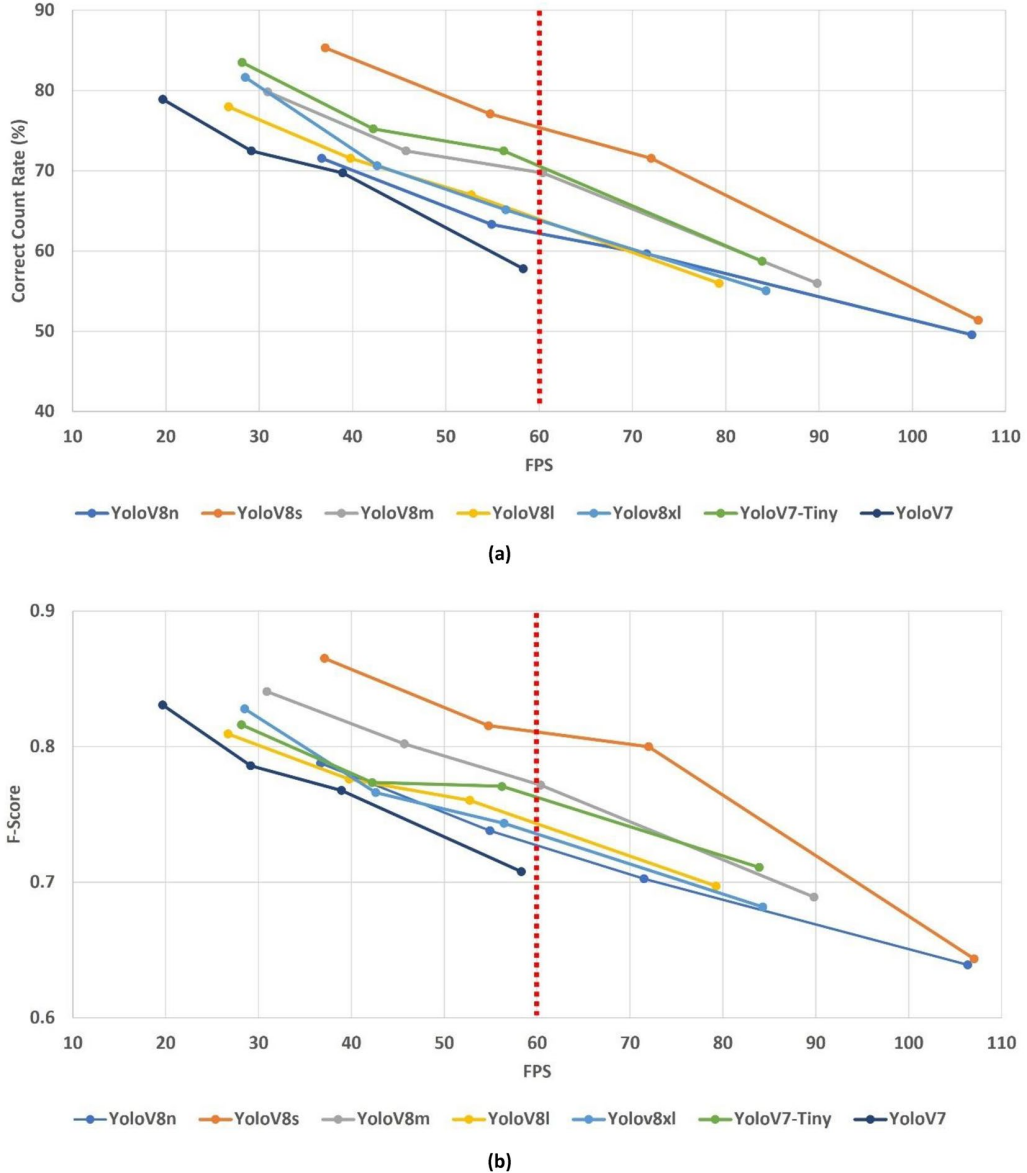
FPS values versus correct count rates (**a**) and F-scores (**b**) for different frame skipping amounts s. Dots from left to right correspond to cases for no frame skipping, FS#1, FS#2, and FS#3, respectively. The real-time threshold is denoted by horizontal red line at 60 FPS.

The three frame skipping approaches other than adaptive frame skipping use predefined intervals for selecting which frames to process. In other words, the rate of increment in the expected FPS value may be calculated to some extent when one of FS#1, FS#2, and FS#3 is applied. On the other hand, this is not the case for adaptive frame skipping in which the skipping decision is made based on the content of the frame. Thus, results associated with the adaptive frame skipping are presented separately from the others. The changes in the correct count rates and F-scores are illustrated in Fig. 5, where the four dots in each line represent the cases for no frame skipping, FS#1, FS#2, and FS#3 from left to right on the horizontal axis.

Different from skipping the frames at a predefined rate, adaptive frame skipping uses the *Nephrops* presence information to determine whether to process more frames. Therefore, it helps improve the overall processing speed when there are intervals with no *Nephrops* detection. In order to illustrate the effectiveness of adaptive frame skipping, the changes in average FPS values and the counting performances for cases with and without frame skipping are given in Table 4.

**Table 4:**
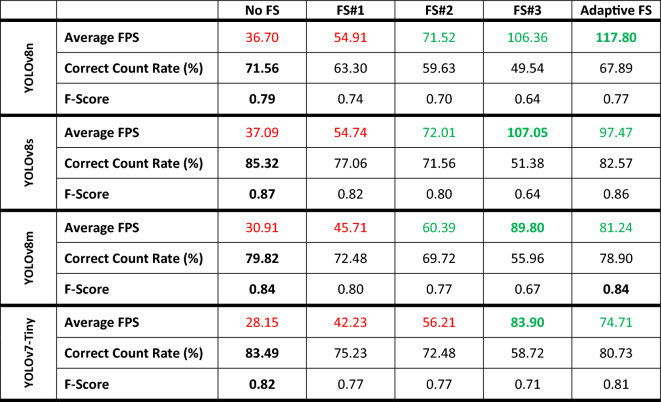
Effects of different frame skipping approaches on speed and counting performances for four YOLO models.

The red and green colors denote the non-real-time and real-time processing speeds, respectively.

Best values are shown in bold.

### Processing speed under constrained power consumption

Introducing an upper limit for power consumption of the edge device affects only the processing speed of the frames. As a result of the experiments, it was observed that setting the power mode to 50 W causes a reduction in the average processing speed between 25 and 30%. In general, the reduction rate is higher for the frame skipping cases with higher amounts of processed frames. In other words, the higher the number of processed frames, the more the overall processing speed is affected by the power limitations.

### Rate of processed frames

The total number of processed frames is the main factor affecting the reported FPS values and this number cannot be predicted in case of adaptive frame skipping. Therefore, the total numbers of processed frames, frames with *Nephrops* detections, and skipped frames are provided to enable a more detailed benchmarking across the object detection models (Table 5).

**Table 5:** Numbers of processed frames, frames with Nephrops detections, and skipped frames for object detection models with adaptive frame skipping applied.

	Video-1	Video-2	Video-3	Video-4	Video-5
YOLOv8n					
No of processed frames	1285	2003	9742	10,327	8348
No of frames with *Nephrops* detections	248	236	1035	706	797
No of skipped frames	2065	3504	17,281	19,080	15,022
YOLOv8s					
No of processed frames	1287	2007	9873	10,722	8392
No of frames with *Nephrops* detections	250	245	1208	1287	859
No of skipped frames	2063	3500	17,150	18,685	14,978
YOLOv8m					
No of processed frames	1289	2007	9865	10,628	8385
No of frames with *Nephrops* detections	254	243	1213	1164	858
No of skipped frames	2061	3500	17,158	18,779	14,985
YOLOv7-Tiny					
No of processed frames	1294	2002	9882	10,602	8395
No of frames with *Nephrops* detections	258	237	1224	1095	872
No of skipped frames	2056	3505	17,141	18,805	14,975

## Discussion

This study exhaustively evaluates numerous training and test time settings for a *Nephrops* counting algorithm utilizing state-of-the-art object detectors and demonstrates that is it possible to provide automatically processed catch information to fishers in real-time using hardware that realistically can be used onboard commercial fishing vessels. The developed image processing system can be transferred to other species and demersal trawl fisheries and can be further developed to handle all relevant species, wanted or unwanted that may enter a given trawl.

### Comparison of object detection models and their settings

All of the seven models in this study were trained for different combinations of the optimizer, batch size, and input image dimension. Instead of providing the results for every single model, an overview of the results is presented for different groups of the training settings (Fig. 3). This allows for summarizing the results and making comparisons between the training settings to determine the most suitable ones. According to Fig. 3, F-score is not an obvious distinguishing factor for counting performance because there is not a significant difference between the F-score values in the different experiments. Comparing the correct count rates, the other counting performance metric, training with the Adam optimizer achieves a better result than SGD in most of the models. In addition, the highest correct count rate and F-score values are obtained with the Adam optimizer, image dimensions of 416 pixels and batch size of 32 images. Therefore, it is possible to conclude that this is a suitable combination for the *Nephrops* counting task, and the models with these settings have been investigated in detail for the following experiments.

It is also possible to perform model-wise comparison using the plots in Fig. 3. On average, the YOLOv7-Tiny model has the best counting performance, but the highest performance overall is achieved by the YOLOv8s model. This means that usage of large and computationally heavy models does not necessarily yield better counting performance in the videos. Such large models may generally be better at making predictions on single images, however within the overall algorithm, they may output some false positive detections due to partial occlusions of the objects. In addition, larger amounts of training epochs and training images may be more useful for a better learning of the larger models. Besides, larger models have longer processing duration of the frames, which is not desired for the current application.

The model type and input image size are the two major factors affecting the overall processing speed, and their associated performances can be compared using the bar graph in Fig. 4. In general, the combination of smaller images and smaller models achieve higher FPS values. However, there are some occasions where the FPS values are higher for larger images. Therefore, it is not possible to expect a negative correlation between them all the time. For a certain model setting, the FPS value and the counting performance may be considered regardless of the setting for input image size.

Based on the two types of performance metrics, four fast and accurate models are selected and their quantitative performances are provided in Tables 2 and 3. The differences in the model performances can be seen precisely in these tables. Among the selected models, YOLOv8s model has the best correct count rate, F-score, and FPS value. Both counting performance metrics of YOLOv8s model outperform those obtained by YOLOv4 which was reported in an earlier study^26^. However, the processing speed averaged over all the test videos is 37.09 FPS, a value that cannot be considered as real-time for the videos considered in this study. As a result, using the state-of-the-art abject detectors on an edge device without any speed enhancement step, it is not possible to achieve real-time processing.

In the related literature, there are no efforts for real-time processing of the underwater videos for catch counting in certain fisheries. Yet, there are some other studies aiming to achieve faster speed in the processing of underwater images. For example, Jahanbakht et al., proposed a lightweight convolutional neural network model for segmentation of the fish on an edge computing device. Even though their model introduces improvements, the associated processing speed is below 5 FPS, a value that cannot be considered real-time^27^. In another study, Soom et al., employed a binary classification model on some hardware with changing computational power to determine presence of fish^40^. They utilized deep learning models to recognize the environmental conditions and frame differencing to detect the foreground objects in the videos. The proposed model reached a maximum processing speed of 45 FPS. These studies do not focus on recognition of a specific fish species which prevents it from being evaluated for a specific fishery.

### Impacts of frame skipping

For the algorithm to be considered as real-time, its corresponding average frame processing speed should be greater than or equal to the frame rate of the input video. Thus, all these models are not capable of producing results in real-time on the edge device when the entire frame sequences are processed (i.e., no frame skipping is implemented). In other words, when all the frames in a given video are processed using the object detection models, the overall delay becomes too much that the catch items cannot be counted in real-time. As a solution to this, four frame skipping ideas were implemented and their respective effects on the performance values explored. It is expected that introducing frame skipping will degrade the counting performance while achieving a faster overall processing. This is shown in Fig. 5 by illustrating the changes in correct count rate and F-score for three frame skipping approaches (FS#1, FS#2, FS#3) together with the case in which all frames are processed. As expected, an increase in the number of frames skipped results in a decrease in counting performance while increasing the average FPS value. None of the models can achieve sufficiently high FPS when FS#1 is applied. Also, the average decrement percentages in the correct count rates and F-scores for FS#1 are 10.75% and 5.74%, respectively. For FS#2, real-time FPS values are obtained only with YOLOv8n, YOLOv8s YOLOv8m models at moderate counting performances. The average rates of decrement in the counting performance in case of FS#2 are 14.66% for correct count rate and 8.46% for F-score. Despite the very high FPS values in case of FS#3, the correct count rates drop below 60% for all the models which cannot be accepted as a sufficiently high value for deployment.

The trade-off mentioned above has been addressed by introducing adaptive frame skipping that allows more frequent processing of the frames whenever a *Nephrops* is detected in the video. In other words, more frames are skipped in the parts of the video with no *Nephrops*. This property enables achieving higher FPS values and counting performance simultaneously. The performance metrics related with all the frame skipping approaches are given in Table 4. Obviously, adaptive frame skipping can considerably improve the FPS value while maintaining the counting performance at relatively high levels. In particular, adaptive frame skipping together with YOLOv8s model can increase speed up the processing nearly 2.6 times and still achieve a correct count rate of 82.57% and an F-score of 0.86. All in all, YOLOv8s model featuring frame skipping and trained with Adam optimizer, image dimensions of 416 pixels, and batch size of 32 achieves the maximum counting performance among all the experimented models and settings. Based on the results obtained, adaptive frame skipping is the most suitable option for the given test videos. The adaptive frame skipping approach is very useful when there are intervals in the video with no *Nephrops*. On the other hand, as the frequency of *Nephrops* instances increase, less frames will be skipped, causing the FPS value to be similar to the no frame skipping case.

### Design and application possibilities for *Nephrops* fisheries

When fishing for *Nephrops*, the limited information on catches in real-time can result in relatively low catch volumes. This lack of information also reduces the sustainability of the fishery, where carbon emissions and seabed disturbances can be unnecessarily high due to poor catch rates. Therefore, providing real-time information about catch rates during fishing is very important for improving the sustainability of fisheries, where such knowledge may be used as a decision tool for optimizing the fishing operation^26^. In other words, such information enables the fishers to improve their targeting behavior, consequently improving catch rates and reducing unnecessary seabed disturbances and carbon emissions.

The automated processing of the videos collected using in-trawl cameras may be accomplished by deep learning-based object detection models. However, such models require computationally powerful hardware to process the video frames in real-time. In addition, this hardware typically consumes a large amount of space and electrical energy. Due to the resource-related restrictions on board fishing vessels, such constraints are not easily resolved. Furthermore, an in-trawl processing system, where video frames are processed underwater without streaming them up to the vessel, will have tighter restrictions. Therefore, it is critical to produce catch statistics, e.g. *Nephrops* catch counts, in real-time while minimizing space and power consumption. Within the scope of this this study, these requirements have been addressed by using an edge device optimized for deep learning applications. The results of the comprehensive experiments conducted on the edge device have revealed the optimal model, its relevant settings, possibility of real-time operation, even under restricted power consumption.

In this context, the change in processing speed mentioned in “Processing speed under constrained power consumption” section provides valuable information. In particular, reducing the maximum allowed power consumption from 60 to 50 W causes a maximum decrement rate of 30%. This implies that if an average FPS value of around 90 is obtained in the max power mode, real-time processing can still be achieved when the power consumption of the device is restricted. This property is particularly useful in cases where the *Nephrops* counting system is expected to be powered by external batteries with limited resources.

### Future improvements in the algorithm

The adaptive frame skipping starts processing consecutive frames whenever a *Nephrops* is detected. Hence, the requirement of more processing emerges intermittently depending on the presence of *Nephrops*. This can be a disadvantage when the *Nephrops* instances are distributed uniformly throughout the video or where the presence of *Nephrops* is high. However, in case of long intervals with no *Nephrops* instances, adaptive frame skipping works sufficiently fast and accurate. If extensive delays are observed in processing when adaptive frame skipping is being used, a workaround can be planned for expediting the processing speed. This may include switching to a faster but relatively inaccurate model or skipping more frames. The processing speed at various temporal densities of *Nephrops* in the videos should be investigated in the future for understanding the system behavior under various real-world scenarios.

Since demersal fisheries typically target multiple species, a deep learning model capable of detecting other species than *Nephrops* would be useful for future applications. In that case, a delay in processing may be introduced in the detection and tracking steps because of the increased number of classes.

During their initial development, the object detection models used in this study are benchmarked on Microsoft Common Objects in Context (MS COCO) dataset that contains objects from 80 different classes. However, this study aims to detect only *Nephrops* instances. Despite the accurate detection of *Nephrops* individuals with the original YOLO models, these models may be revised and modified to reduce their computational needs and still be appropriate for *Nephrops* detection. Such an updated model may generate the output in a shorter time and help achieve real-time processing with reduced power consumption.

## Conclusion

This study includes detailed experiments on searching for an appropriate object detection model and its training settings for automatically counting the *Nephrops* catch items in real time during demersal trawling. For this purpose, the videos collected using an in-trawl camera are processed on an edge device that is suitable to be used on board fishing vessels. Seven different object detection models with varying complexities have been involved in the experiments and four frame skipping approaches have been analyzed to achieve real-time processing speeds with high counting performance. It has been observed that the highest correct count rate is achieved when YOLOv8s model is trained using Adam optimizer, input image size of 416, and batch size of 32. However, there are other models with respective settings that may produce similar outputs in real-time while achieving satisfactory counting performance. In addition, application of the adaptive frame skipping has introduced significant improvement in average processing speed at an expense of minor degradation in correct count rate.

The results indicate that the proposed method can be used for real-world applications and make demersal trawling in general and for current studies results the *Nephrops* fishery a more informed and targeted operation and make trawls as a fishing method more sustainable. In particular, this study may help mitigating some of the fundamental problems associated with demersal trawl fisheries such as seabed impact, excessive fuel emissions, and high amounts of bycatch. Therefore, the proposed approach is a promising tool for improving the sustainability and transparency of demersal trawling for current and future trawl fisheries.

### Supplementary Information

Supplementary Information.

## Data Availability

Publicly available datasets were analyzed in this study. This data can be found here: 10.11583/DTU.21769442.
