# Supplementary material for: Edge computing based real-time Nephrops (Nephrops norvegicus) catch estimation in demersal trawls using object detection models
Source: Sci Rep. 2024 Apr 25;14:9481. doi: 10.1038/s41598-024-60255-8 (PMC11045813; doi:10.1038/s41598-024-60255-8)
Supplement: Supplementary file 1 — Supplementary Information. [file 41598_2024_60255_MOESM1_ESM.docx]

**Supplementary Information-1**

The distributions of the *Nephrops* instances throughout the test videos are critical for assessing the performance of the proposed methodology. They particularly affect the frame processing frequency for the adaptive frame skipping approach hence the associated FPS value. Therefore, in this appendix, the information for total number of frames and the number of frames containing at least one *Nephrops* are provided (Table S1.1) as well as the details about the individual *Nephrops* instances in each video (Table S1.2). These two types of information are useful for understanding the density and distribution of the *Nephrops* presence in the test videos.

**Table S1.1.** Counts of total frames and the frames with *Nephrops* presence

|  | **Video-1** | **Video-2** | **Video-3** | **Video-4** | **Video-5** |
| --- | --- | --- | --- | --- | --- |
| **Total frames** | 3350 | 5507 | 27023 | 29407 | 23370 |
| **Frames with *Nephrops*** | 259 | 226 | 1053 | 1420 | 712 |
| **Ratio of frames with *Nephrops* (%)** | 7.73 | 4.10 | 3.90 | 4.83 | 3.05 |

**Table S1.2.** Beginning and end frames of all the *Nephrops* individuals in the test videos

| ***Nephrops* No** | **Video-1** | | **Video-2** | | **Video-3** | | **Video-4** | | **Video-5** | |
| --- | --- | --- | --- | --- | --- | --- | --- | --- | --- | --- |
|  | **From** | **To** | **From** | **To** | **From** | **To** | **From** | **To** | **From** | **To** |
| **1** | 1 | 49 | 55 | 93 | 1516 | 1535 | 356 | 384 | 202 | 224 |
| **2** | 97 | 215 | 175 | 203 | 1607 | 1624 | 1890 | 1895 | 188 | 287 |
| **3** | 1638 | 1717 | 982 | 994 | 1662 | 1687 | 2501 | 2511 | 388 | 444 |
| **4** | 2207 | 2218 | 2103 | 2136 | 1905 | 1940 | 2643 | 2646 | 780 | 786 |
| **5** | - | - | 4398 | 4424 | 2839 | 2877 | 4152 | 4206 | 857 | 860 |
| **6** | - | - | 5039 | 5128 | 2863 | 2903 | 4529 | 4681 | 1279 | 1345 |
| **7** | - | - | - | - | 3403 | 3410 | 6900 | 6953 | 3203 | 3234 |
| **8** | - | - | - | - | 3723 | 3736 | 8238 | 8244 | 5116 | 5133 |
| **9** | - | - | - | - | 4412 | 4428 | 8294 | 8311 | 5890 | 5895 |
| **10** | - | - | - | - | 5790 | 5799 | 8652 | 8671 | 6071 | 6102 |
| **11** | - | - | - | - | 6179 | 6215 | 8999 | 9029 | 6565 | 6576 |
| **12** | - | - | - | - | 7738 | 7760 | 9104 | 9174 | 6963 | 6977 |
| **13** | - | - | - | - | 7892 | 7914 | 9330 | 9394 | 7378 | 7384 |
| **14** | - | - | - | - | 13150 | 13167 | 9709 | 9731 | 7936 | 8014 |
| **15** | - | - | - | - | 13141 | 13247 | 10176 | 10198 | 9263 | 9268 |
| **16** | - | - | - | - | 14346 | 14353 | 10195 | 10218 | 9418 | 9433 |
| **17** | - | - | - | - | 15193 | 15206 | 10208 | 10219 | 11395 | 11476 |
| **18** | - | - | - | - | 15530 | 15552 | 10282 | 10300 | 11581 | 11607 |
| **19** | - | - | - | - | 17186 | 17187 | 10293 | 10381 | 12981 | 12987 |
| **20** | - | - | - | - | 17122 | 17218 | 10299 | 10379 | 13154 | 13162 |
| **21** | - | - | - | - | 19587 | 19598 | 10654 | 10766 | 13934 | 14039 |
| **22** | - | - | - | - | 19639 | 19655 | 10782 | 10789 | 15072 | 15079 |
| **23** | - | - | - | - | 20044 | 20203 | 12570 | 12582 | 21292 | 21307 |
| **24** | - | - | - | - | 20820 | 20828 | 13462 | 13470 | - | - |
| **25** | - | - | - | - | 21779 | 21813 | 14347 | 14361 | - | - |
| **26** | - | - | - | - | 22176 | 22192 | 17040 | 17066 | - | - |
| **27** | - | - | - | - | 22145 | 22224 | 18618 | 18716 | - | - |
| **28** | - | - | - | - | 22259 | 22284 | 19195 | 19226 | - | - |
| **29** | - | - | - | - | 22858 | 22877 | 20386 | 20467 | - | - |
| **30** | - | - | - | - | 22897 | 22919 | 20720 | 20754 | - | - |
| **31** | - | - | - | - | 22912 | 22922 | 20755 | 20783 | - | - |
| **32** | - | - | - | - | 23149 | 23159 | 21878 | 21910 | - | - |
| **33** | - | - | - | - | 23270 | 23303 | 22072 | 22091 | - | - |
| **34** | - | - | - | - | 25391 | 25446 | 23923 | 23952 | - | - |
| **35** | - | - | - | - | 26519 | 26541 | 24200 | 24252 | - | - |
| **36** | - | - | - | - | 26522 | 26533 | 25183 | 25265 | - | - |
| **37** | - | - | - | - | - | - | 25396 | 25432 | - | - |
| **38** | - | - | - | - | - | - | 25416 | 25423 | - | - |
| **39** | - | - | - | - | - | - | 27080 | 27124 | - | - |
| **40** | - | - | - | - | - | - | 29117 | 29120 | - | - |
